# Evaluation of novel LCI CAD EYE system for real time detection of colon polyps

**DOI:** 10.1371/journal.pone.0255955

**Published:** 2021-08-26

**Authors:** Helmut Neumann, Andreas Kreft, Visvakanth Sivanathan, Fareed Rahman, Peter R. Galle

**Affiliations:** 1 Department of Interdisciplinary Endoscopy, I. Medizinische Klinik und Poliklinik, University Hospital, Mainz, Germany; 2 GastroZentrum Lippe, Bad Salzuflen, Germany; 3 Institute of Pathology, University Hospital, Mainz, Germany; University of Craiova, ROMANIA

## Abstract

**Background:**

Linked color imaging (LCI) has been shown to be effective in multiple randomized controlled trials for enhanced colorectal polyp detection. Recently, artificial intelligence (AI) with deep learning through convolutional neural networks has dramatically improved and is increasingly recognized as a promising new technique for enhancing colorectal polyp detection.

**Aim:**

This study aims to evaluate a newly developed computer-aided detection (CAD) system in combination with LCI for colorectal polyp detection.

**Methods:**

First, a convolutional neural network was trained for colorectal polyp detection in combination with the LCI technique using a dataset of anonymized endoscopy videos. For validation, 240 polyps within fully recorded endoscopy videos in LCI mode, covering the entire spectrum of adenomatous histology, were used. Sensitivity (true-positive rate per lesion) and false-positive frames in a full procedure were assessed.

**Results:**

The new CAD system used in LCI mode could process at least 60 frames per second, allowing for real-time video analysis. Sensitivity (true-positive rate per lesion) was 100%, with no lesion being missed. The calculated false-positive frame rate was 0.001%. Among the 240 polyps, 34 were sessile serrated lesions. The detection rate for sessile serrated lesions with the CAD system used in LCI mode was 100%.

**Conclusions:**

The new CAD system used in LCI mode achieved a 100% sensitivity per lesion and a negligible false-positive frame rate. Note that the new CAD system used in LCI mode also specifically allowed for detection of serrated lesions in all cases. Accordingly, the AI algorithm introduced here for the first time has the potential to dramatically improve the quality of colonoscopy.

## Introduction

Colonoscopy is the gold standard for the detection of colorectal adenomas, which are considered to be the precursor lesions of colorectal cancer [1]. Despite being the gold standard, the efficacy of colonoscopy is reduced by a significant miss rate regarding adenomas and the high variability in adenoma detection rates among endoscopists [2]. In addition, sessile serrated lesions, often occurring as slightly elevated lesions with indistinctive borders, significantly contribute to missed lesions and interval cancer [3]. In recent years, various image-enhanced endoscopy systems have been shown to be effective for reducing adenoma miss rates, thereby significantly improving the quality of colonoscopy examinations [4, 5]. However, these systems have only been shown to be effective in the hands of so-called experts; they have failed to be effective in community practice [6, 7].

More recently, computer-assisted imaging modalities have been introduced, allowing for the detection of colorectal lesions based on artificial intelligence (AI) algorithms comprising convolutional neuronal networks [8]. Although early data are promising, those systems are either not yet commercially available or are not produced by endoscopy manufacturing companies, thereby only offering limited access to the global endoscopy market and specifically to the community practice. In addition, none of the systems have yet been shown to be effective in detecting sessile serrated lesions.

In this paper, we present, for the first time, the results of a new AI system for real-time colorectal polyp detection, including sessile serrated lesions, which is CE-certified. Moreover, the system is to date the only one that is introduced by an endoscopy manufacturing company, making it specifically available even for community practice.

## Methods

For the purpose of this study, a new AI system (CAD EYE, Fujifilm, Tokyo, Japan) based on a convolutional neural network was trained and validated using more than 500 videos of 2,000 histologically confirmed polyps (Table 1). The training set included 1,063 polyps with high-definition white light and 938 polyps with linked color imaging (LCI, Fujifilm, Tokyo, Japan). A total of more than 200,000 frames of the polyps was extracted from these videos and manually compiled by expert endoscopists.

**Table 1:** Clinicopathological features of the training set.

Parameter	N
Videos for validation set	500
Histologically confirmed polyps within the dataset	2000
Polyps imaged with high-definition white light	1063
Polyps imaged with linked color imaging	938

For the validation phase, 240 polyps within fully recorded endoscopy videos with LCI mode, covering the entire spectrum of adenomatous histology, were used (Table 2). Inclusion criteria were full endoscopy withdrawal videos with LCI of patients undergoing screening or surveillance endoscopy. Exclusion criteria were defined as non-adequate bowel preparation with a BBPS of < 6 and no full-length withdrawal in LCI mode. Of the included 240 polyps of the validation set, 59 were hyperplastic polyps, 82 were traditional adenomas, 34 were sessile serrated lesions, 2 were early cancers, and 63 were non-neoplastic lesions, such as inflammatory polyps. Of note, video clips were not cut; the full-length withdrawal videos were evaluated without any preprocessing. One experienced endoscopist with experience of > 10,000 endoscopies has judged the recordings. Analysis was performed with the human eye.

**Table 2:** Clinicopathological features of polyps of the validation set.

Histology	N
Hyperplastic polyps	59
Traditional adenomas	82
Sessile serrated lesions	34
Early cancers	2
non-neoplastic lesions (e.g. inflammatory polyps)	63

Here, the Skip Connections Net model type was used for the CNN. Its architecture consisted of multiple convolution layers that performed a 3 × 3 or 1 × 1 convolution process. For final output, a softmax function was used that sets the sum of multiple values to 1.0. For training, the ratio across modalities was set at 1:1 for white-light imaging (WLI) and linked color imaging (LCI) for detection. An adequate number of frames without lesions was also included. Hold-out validation (i. e. different polyps used for training and testing) was used.

### Description of CAD EYE

The CAD EYE (Fujifilm, Tokyo, Japan) is a new AI system developed for the detection of colorectal polyps (S1 Video). The AI is integrated in an external box (EX-1, Fujifilm, Tokyo, Japan) that is placed on the normal video processor and connected with a DVI input. The box itself is connected to a standard video monitor. The CAD EYE is activated by the endoscopist by pushing a button on the handle of the endoscope. The system is fully compatible with all video processors and colonoscopes from the ELUXEO^TM^ 7000/ELUXEO^TM^ Lite EP-6000 series. The CAD EYE has a specific graphic user interphase, highlighting the polyp by placing a colored box around it. In addition, a detection sound is emitted when a suspicious polyp is detected. Finally, a visual assist circle lights up in the direction where the polyp is detected, thereby facilitating identification of the polyp by the endoscopist. EX-1 comes with an integrated video recording function that allows the storage of all videos on an internal hard drive.

### Statistics and ethical considerations

To assess sensitivity, true-positive lesions were defined as polyps that were detected by the AI with a stable detection box, lasting for at least 1 s and with an accompanying sound (S1 Video). Otherwise, the lesion was defined as false negative. To assess the number of false-positive frames, endoscopy videos were completely evaluated on withdrawal of the scope from the cecum; all frames where the AI algorithm detected a lesion were judged as true-positive or false-positive lesions. The number of false-positive frames was calculated by dividing the number of false-positive frames by the total number of frames in a full-length withdrawal video. The study was approved by the ethical committee of Rhineland-Palatinate and registered at ClinicalTrials.gov (NCT04339855). All data/samples were fully anonymized before assessment.

### Role of funding source

HN developed the study protocol independent of the study sponsor. Data collection was performed at local sites with a study coordinator supplied by the sponsor. The investigators had full access to all the data and performed all data analysis and data interpretation independently. The sponsor had no role in writing the report. The investigators had final responsibility for the decision to submit the report for publication.

## Results

**Fig 1:**
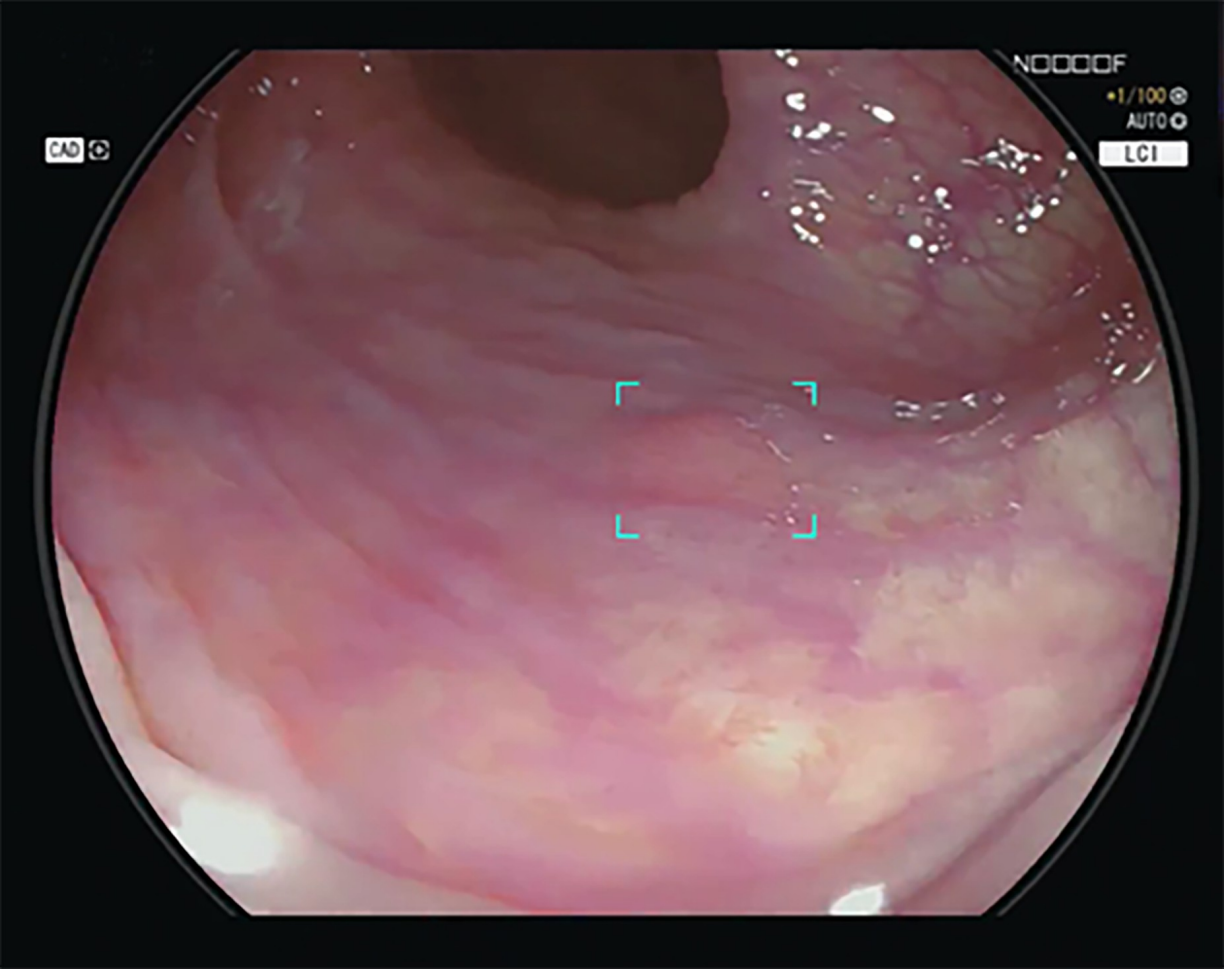
The AI system highlights the polyp by placing a colored box around it. In addition, the detection is accompanied by a specific sound. Here, the polyp is located in the central portion of the endoscopic image. Therefore, the specific visual assist circle is not activated compared with the polyp in Fig 2.

**Fig 2:**
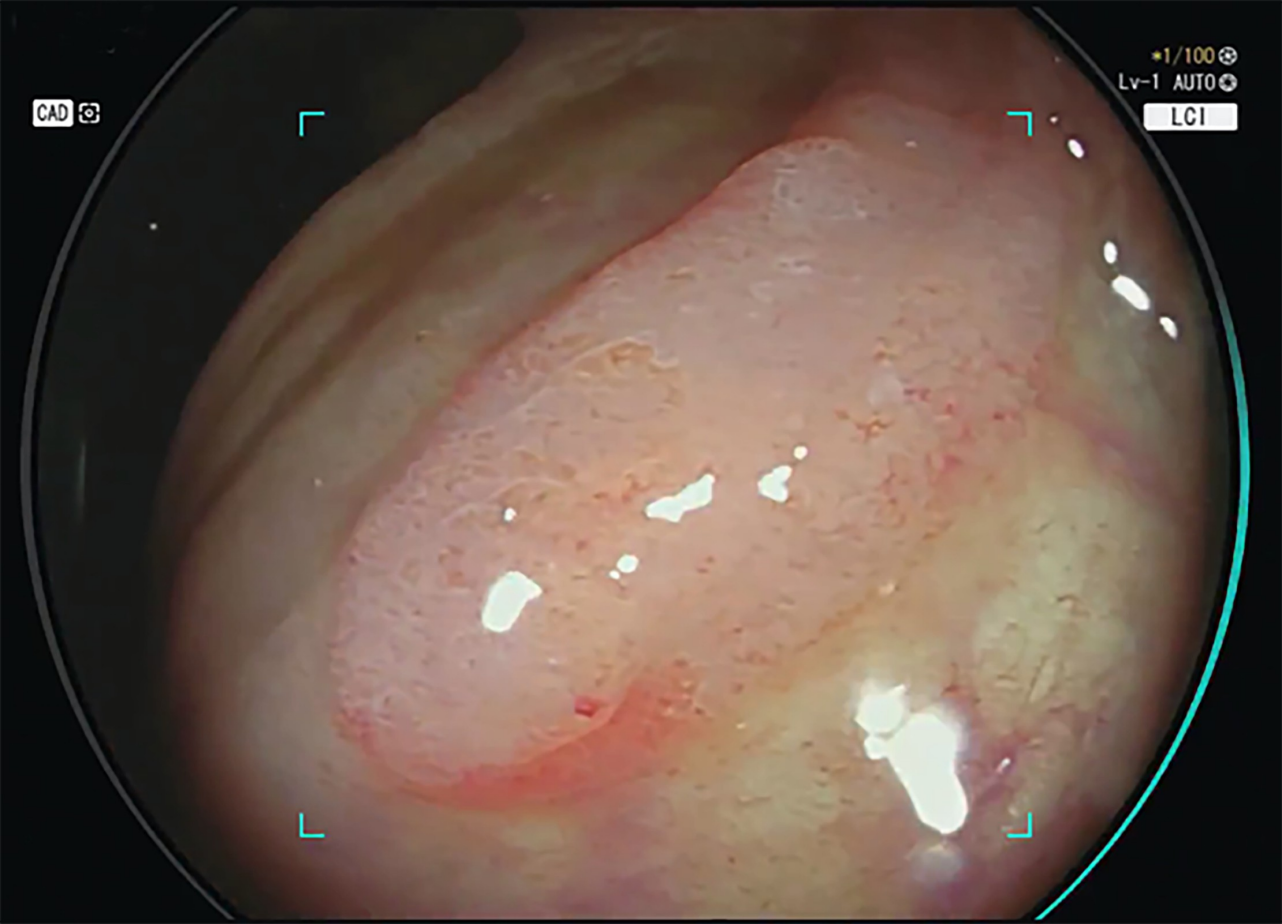
The specific graphic user interphase of the CAD EYE system also highlights the direction in which the polyp has been detected. Here, the polyp is directly located behind one colonic fold of the ascending colon and might therefore be easily missed. The full-length sequence is available in the S1 Video, especially highlighting the position of the polyp in the ascending colon.

The new CAD EYE system used in the LCI mode could process at least 60 frames per second, thereby allowing for real-time video analysis (Figs 1 and 2; S1 Video). Sensitivity (true-positive rate per lesion) was 100%, with no lesion being missed. The calculated false-positive frame rate for one entire colonoscopy video was 0.001%. Among the 240 polyps, 34 were sessile serrated lesions. The detection rate for sessile serrated lesions with the CAD system used in the LCI mode was 100%.

## Discussion

The new CAD system used in LCI mode achieved 100% sensitivity per lesion and a negligible false-positive frame rate. Of note, the new CAD system used in LCI mode also specifically allowed for the detection of sessile serrated lesions in all cases in our study.

Colonoscopy is accepted as the most powerful screening modality for colorectal cancer and its precursor lesions [9]. Since the introduction of colorectal cancer screening programs, a steady decrease in the incidence of colorectal cancer has been observed [10]. However, it is also well accepted that interval cancer after colonoscopy can occur and that a significant number of lesions can be missed by standard colonoscopy [11, 12].

Multiple studies have evaluated the potential of various image-enhanced endoscopy systems to improve adenoma detection rates. Although some studies have shown the benefits of image-enhanced endoscopy systems, they fail to prove their effectiveness in community practice in general [6, 7]. One important reason for this issue might include the limited time of healthcare professionals to become familiar with a new technology to face the learning curve until a new technology can be of beneficial effects. From all of the currently available image-enhanced endoscopy systems, the most promising literature comes from large, randomized control trials addressing the LCI technology (LCI, Fujifilm, Tokyo, Japan), which is included in all video processors of the ELUXEO line [13, 14]. Unlike other imaging modalities, which were initially developed only for characterization of lesions (i.e., optical chromoendoscopy techniques), LCI was specifically designed to foster detection of slightly elevated lesions by enhancing red color tones while retaining the natural color of the tissue. Since our study aims to specifically detect sessile serrated lesions, we trained the AI algorithm with the LCI technology.

Various factors have been discussed as being responsible for missed lesions and interval cancer, including inadequate bowel preparation, polyp morphology, and location. In addition, human factors such as experience, fatigue, and distraction are crucial [15]. The significance of endoscopists’ experience has been shown in multiple studies, describing adenoma detection rates with a wide range between 20% and 60% [16]. In this context, AI-driven systems offer the potential to exclude human factors.

Recently, an AI system was trained on endoscopy videos, achieving a sensitivity of 99.7% and a false-positive frame rate of less than 1% [17]. For each of the polyps used in the study, a video clip was cut starting 5 s before polyp appearance and ending when the snare or biopsy forceps appeared. This might have caused a potential major bias, as AI systems generally consider multiple variables for the learning process. In this context, the AI system might have recognized that polyps are specifically occurring after 5 s. The endpoint was the appearance of a snare or biopsy for resection. For endoscopic resection, the polyp is mostly placed at the six o’clock position and close to the endoscope. True-positive lesions in the study were considered in the study when the polyp was located in at least one frame. Taking all the above-mentioned characteristics into consideration, one can expect a major bias for the AI system regarding polyp detection.

To exclude this bias, we defined true-positive lesions as polyps that have been detected by the AI lasting for at least 1 s and an accompanying sound. In addition, full endoscopy videos were evaluated to best reflect real-life situations. Videos were chosen to mitigate the apparent operator-related bias when evaluating an advanced imaging modality. Obviously, an endoscopist cannot be blinded to the innovative technology that has been studied.

Many other groups have evaluated different AI algorithms for the detection of lesions. However, none of those systems have been approved by the authorities or are commercially available. A detection rate greater than 90% was achieved by Karkanis et al. for the detection of adenomatous polyps [18]. However, the AI system was only based on still images, and translation to clinical medicine was not possible. Fernández-Esparrach et al. reported on the efficacy of a computer-assisted algorithm for polyp detection [19]. Although the authors tested the algorithm on videos from routine colonoscopies, they only achieved 70% sensitivity. Recently, Misawa and coworkers introduced a novel computer-aided detection (CAD) system that detected 95% of test polyps, with a false-positive detection rate of 60% [20].

Here, we report for the first time on a computer-assisted diagnostic modality allowing for real-time detection of colorectal polyps during routine colonoscopy. Although real-life videos were used, containing residual stool components and other artifacts, the AI algorithm detected all colonic polyps with a negligible false-positive frame rate. Note that while sessile serrated lesions are considered to play a crucial role in interval cancer and contribute to a significant number of missed lesions during colonoscopy, the AI algorithm detected all sessile serrated lesions in the present study.

The clinical potential of the novel AI algorithm is therefore exceedingly high. The system is compatible with normal endoscopy equipment and can be used *ad hoc*. The learning curve, which is always considered a critical part of the introduction of a novel technique to endoscopy, is not relevant here, as the AI algorithm assists all endoscopists, irrespective of their experience level. Accordingly, the AI algorithm introduced here for the first time has the potential to dramatically improve the quality of colonoscopy, especially in community practice. Further, we expect incorporation in our routine clinical practice within a very short period of time.

## Supporting information

S1 Video The video highlights the graphic user interphase and the detection sound of the new AI algorithm for automatic polyp detection and the detection of a Paris-type IIa lesion in the ascending colon, which is hidden behind a colonic fold. (WMV)
